# Reduction in all-cause otitis media-related outpatient visits in children after PCV10 introduction in Brazil

**DOI:** 10.1371/journal.pone.0179222

**Published:** 2017-06-08

**Authors:** Ana L. Sartori, Ruth Minamisava, Ana L. Bierrenbach, Cristiana M. Toscano, Eliane T. Afonso, Otaliba L. Morais-Neto, José L. F. Antunes, Elier B. Cristo, Ana Lucia Andrade

**Affiliations:** 1 Department of Community Health, Institute of Tropical Pathology and Public Health, Federal University of Goiás, Goiânia, Brazil; 2 Institute of Health Sciences, Federal University of Mato Grosso, Sinop, Brazil; 3 School of Nursing, Federal University of Goiás, Goiânia, Brazil; 4 School of Medicine, Federal University of Goiás, Goiânia, Goiás, Brazil; 5 School of Public Health, University of São Paulo, São Paulo, Brazil; 6 Advisor of the Secretariat of Health, São Paulo, São Paulo, Brazil; Public Health England, UNITED KINGDOM

## Abstract

Few studies have reported the effect of 10-valent pneumococcal conjugate vaccine (PCV10) on otitis media (OM) in infants. In particular, no population-based study in upper-middle income countries is available. In 2010, Brazil introduced PCV10 into its routine National Immunization Program using a 3+1 schedule. We measured the impact of PCV10 on all-cause OM in children. An interrupted time-series analysis was conducted in Goiânia/Brazil considering monthly rates (per 100,000) of all-cause OM outpatient visits in children aged 2–23 months. We used case-based data from the Outpatient Visits Information System of the Unified Health System coded for ICD-10 diagnosis for the period of August/2008 to July/2015. As a comparator, we used rates of outpatient visits due to all-other causes. The relative reduction of all-cause OM and all-other causes of outpatient visits were calculated as the difference between the predicted and observed cumulative rates of the PCV10 post-vaccination period. We then subtracted the relative reduction of all-other causes of outpatient visits from all-cause OM to obtain the impact of PCV10 on OM. In total, 6,401 OM outpatient visits were recorded in 4,793 children aged 2–23 months. Of these, 922 (19.2%) children had more than one OM episode. A significant reduction in all-cause OM visits was observed (50.7%; 95%CI: 42.2–59.2%; p = 0.013), while the reduction in visits due to all-other causes was 7.7% (95% CI 0.8–14.7%; p<0.001). The impact of PCV10 on all-cause OM was thus estimated at 43.0% (95%CI 41.4–44.5). This is the first study to show significant PCV10 impact on OM outpatient visits in infants in a developing country. Our findings corroborate the available evidence from developed countries.

## Introduction

Otitis media (OM) is one of the main reasons for seeking healthcare services and receiving an antibiotic prescription during childhood in both developed and developing countries [1, 2]. Acute otitis media (AOM) might progress with chronic complications and impairment in quality of life resulting in significant burden for the children and their families [3], and being the most important cause of hearing loss in the world [4]. *Streptococcus pneumoniae* (*S*. *pneumoniae*) and *Haemophilus influenzae* (*H*. *influenzae*) are the most common bacterial causes of AOM in children [5, 6]. Evidence shows that 80% of children up to 3 years of age experience one or more episodes of AOM [7]. Health and economic burden associated to AOM is thus significant [8].

Few studies have evaluated the impact of pneumococcal conjugate vaccination (PCV) in *S*. *pneumoniae* OM, mainly due to difficulties in obtaining middle ear fluid cultures allowing for etiologic diagnosis [9–11]. Therefore, all-cause OM has been the preferred outcome to evaluate the impact of PCV vaccination. Results of efficacy randomized trials of 7-valent PCV pneumococcal conjugate vaccine (PCV7) to prevent all-cause AOM showed modest beneficial effects in healthy infants and no benefit for preventing further episodes in high-risk infants [12, 13]. A systematic review of eight observational studies reported an average reduction in OM visit rates of 19% (range 7–48%) after PCV7 introduction [12]. In the US, PCV7 and 13-valent PCV (PCV13) were able to reduce rates due to all-cause OM from 20% to 43% after the introduction of vaccination into routine immunization programs [2, 14–17]. Similar result was found in Iceland with PCV10, where a 26% reduction in AOM was reported in a recent epidemiological retrospective analysis of children admitted to the hospital and those treated as outpatients [18]. Healthcare in Brazil is provided by both public and private sectors. Public healthcare services are provided by the National Unified Health System (SUS), which offers free of charge, and universal access to all population. Healthcare management is decentralized, with municipalities being responsible for primary care services [19]. SUS accounts for about 77% of outpatient consultations in the country [20]. In the last decade, selected Brazilian municipalities have implemented an electronic information system of outpatient visits within the SUS. Goiânia is one of such municipalities, where individual data for outpatient visits is available at the Municipal Health Department. The main diagnosis of each medical visit is coded in the using the International Classification of Diseases 10^th^ revision (ICD-10).

In 2010, Brazil introduced the PCV10 into its routine National Immunization Program (NIP), in a three-dose schedule administered at 2, 4 and 6 months of age, plus a booster at 12–15 months [21]. PCV10 contains serotypes 1, 5 and 7F, in addition to the serotypes included in PCV7 (4, 6B, 9V, 14, 18C, 19F, 23F), and is conjugated to nontypeable *Haemophilus influenzae* (NTHi) protein D [22].

The impact of PCV10 vaccination on rates of pneumonia hospitalizations, invasive pneumococcal disease (IPD), as well as its effectiveness on nasopharyngeal carriage has been well documented in Brazil [23–27]. Nonetheless, despite its introduction in several countries, few studies have reported the effects of PCV10 on OM [18, 28, 29]. So far, no population-based study has reported the impact of PCV10 on otitis in an upper-middle income country.

Considering the availability of an information system, which allows the use of population based primary care outpatient visits within SUS in a large metropolis in Brazil, we conducted an interrupted time-series analysis to evaluate the impact of PCV10 on rates of outpatient visits for all-cause OM in children, from the SUS perspective, in Brazil.

## Methods

### Study area and population

The study included medical outpatient visits in children aged 2 to 23 months, i.e., children targeted for vaccination, from August 2008 through July 2015 in Goiânia municipality, Brazil. Despite currently being an upper-middle income country, there are important socio-economic inequalities across the country [30]. Goiânia is located in the Central-West Region of Brazil, which has shown the highest income inequality of the country [31]. The municipality has approximately 1.3 million inhabitants comprising 85,000 children under 5 years of age [32]. Almost 70% of the municipality´s population uses SUS [20]. Primary care facilities in the study area are covered by the Brazilian Family Health Strategy, which covers mainly areas of low-income population who usually cannot afford private health care insurance [19]. Lower income population is eligible for *Bolsa Família*, a comprehensive conditional cash transfer program which has improved health care access in this population in recent years [33].

### Study design and data source

An interrupted time-series analysis was conducted to measure the impact of PCV10 on monthly rates of all-cause OM outpatient visits. We used the electronic Outpatient Visit Information System (OVIS), in which all outpatient visits occurring in SUS facilities in Goiânia are recorded in real time within a software intranet built exclusively for administrative and clinical management purposes for the Municipal Health Department. Therefore, access to OVIS is not public, although the database may be made available by the Municipal Health Department upon request, particularly for research purposes. The OVIS was implemented in 2004, firstly in few pilot primary care facilities, and in 2008 throughout the primary care units in the municipality. In 2010, the system was expanded to also include emergency room visits. Information about the patient, including the medical diagnosis justifying the visit are recorded online by the attending physician considering ICD-10 codes [34]. During the study period, OVIS’s covered 91 healthcare facilities with pediatrics attendance. To avoid considering data from healthcare units, which reported data to OVIS in a non-consistent manner, for the present analysis we used data from 75 facilities, which reported data throughout the study period on a regular basis. The 16 health care centers not include in this study are those which were included in OVIS only in 2009, not being major service providers to the community, as the cases of OM in children aged 2–23 months in the period in which they reported data account for only 5.5% of the total OM cases reported in this period. We only considered outpatient visits occurring in primary care, and not emergency room visit, as these were also only incorporated into the OVIS in 2010.

Yearly population estimates of children aged 2 to 23 months used to estimate rates were obtained from the Brazilian Census conducted in 2000 and 2010 from the National Institute of Geography and Statistics. Monthly population estimates were obtained by interpolation based on data from both Brazilian Census [35].

### PCV10 vaccination

The intervention of interest was PCV10 introduction, which occurred in the municipality of Goiânia in mid-June 2010.

For the interrupted time-series analysis, we defined pre-vaccination (August 2008 to July 2010) and post-vaccination (August 2011 to July 2015) periods. PCV10 coverage rates in Goiânia increased rapidly after its introduction, achieving stable rates of 90–95% since 2011 [36]. We thus considered the period from August 2010 to July 2011 as a transition period and we excluded it from the analysis.

### Outcome and comparison group

The outcome of interest was the rate of all-cause OM outpatient visits. All-cause OM was defined by any diagnosis justifying the medical visit, as coded by the following ICD-10 codes: H65 (nonsuppurative otitis media), H66 (suppurative and unspecified otitis media) or H67 (otitis media in diseases classified elsewhere). Any OM visit identified within 21 days of a previous OM visit in the same individual was assumed as the same episode of disease [37], and therefore excluded from the analysis.

For comparison purposes, we considered rates of outpatient visits due to all-other causes, except ICD-10 codes for diseases of the respiratory (Chapter X) and for diseases of the ear and mastoid process (Chapter VIII) during the study period. These were excluded as they are likely to be affected by the intervention in question (PCV vaccination) and therefore are not suitable to serve as outcome controls in such analysis.

### Data analysis

We used deterministic record linkage algorithm to identify additional visits for the same patient, and also to identify duplicated records of the same visit, which were then excluded [38, 39]. After that, we generated a database of monthly incidence rates and, all the data were then analyzed anonymously.

Monthly rates were calculated for all-cause OM visits and visits due to all-other causes using the number of outpatient visits as the numerator, and monthly estimates of children population aged 2 to 23 months as the denominator, then multiplying the result by 100,000 (S1 Dataset).

Trends in monthly rates for all-cause OM visits and visits due to all-other causes were estimated. The time-series comprised 72 months of observation, being 24 in the pre-vaccination and 48 in the post-vaccination period. Secular trend was confirmed by the signal test (Cox-Stuart) and linear regression. Kruskal-Wallis test was used to ascertain seasonality. Rates in the pre-vaccination period were used to predict rates of all-cause OM and other-causes in the post-vaccination period. The interrupted time-series analysis was based on exponential smoothing Holt-Winters additive model [40], to control for pre-existing trends and seasonal variations. The explanatory variables in the model were calendar month (to control for seasonality), and linear trend over time (to control for pre-existing trends). The model provides an exponentially weighted moving average of all observed values in the pre-vaccination period. The premise is that the greater weights are attributable to recent observations in the pre-vaccination period; the recent-past has a more pronounced influence to predict the future. Based on pre-vaccination observed monthly rates of all-cause OM visits and visits due to any-other causes we estimated monthly-predicted outpatient visits rates per 100,000 children for the post-vaccination period. Relative reduction in rates was calculated as the observed rates divided by the predicted cumulative rates in the post-vaccination period minus one. The impact of PCV10 vaccination was obtained from subtracting the relative reduction of outpatient visits due to any-other causes from the relative reduction in rates of all-cause OM and their corresponding 95% confidence interval (95%CI) and p-values (<0.05). Data management was performed using STATA v. 12.0. We used R software for all data analysis and graphics.

### Ethical approval

This study was approved by the Ethics Committee from the Federal University of Goiás, Goiânia, Brazil in 2014 (protocols #162.532 and #1.374.719).

## Results

Overall, 6,401 outpatient visits due to OM in children aged 2 to 23 months were recorded in OVIS in Goiânia from August 2008 to July 2015. We excluded 686 (10.7%) duplicate records of the same OM episode, resulting in a total of 5,715 episodes occurring in 4,793 children which were considered in the analysis. The majority of episodes (53.4%) occurred in children aged 2 to 11 months. A total of 922 children (19.2%) presented more than one otitis episode during the study period. Median age of the first otitis episode was 11 months (interquartile range [IQR: 7–16]), and 54.9% (3,135) of the episodes occurred in boys. Among 5,715 OM episodes, 2,673 (46.8%), 2,985 (52.2%), and 57 (1.0%) visits corresponding to H65, H66, and H67 ICD-10 codes, respectively.

**Fig 1:**
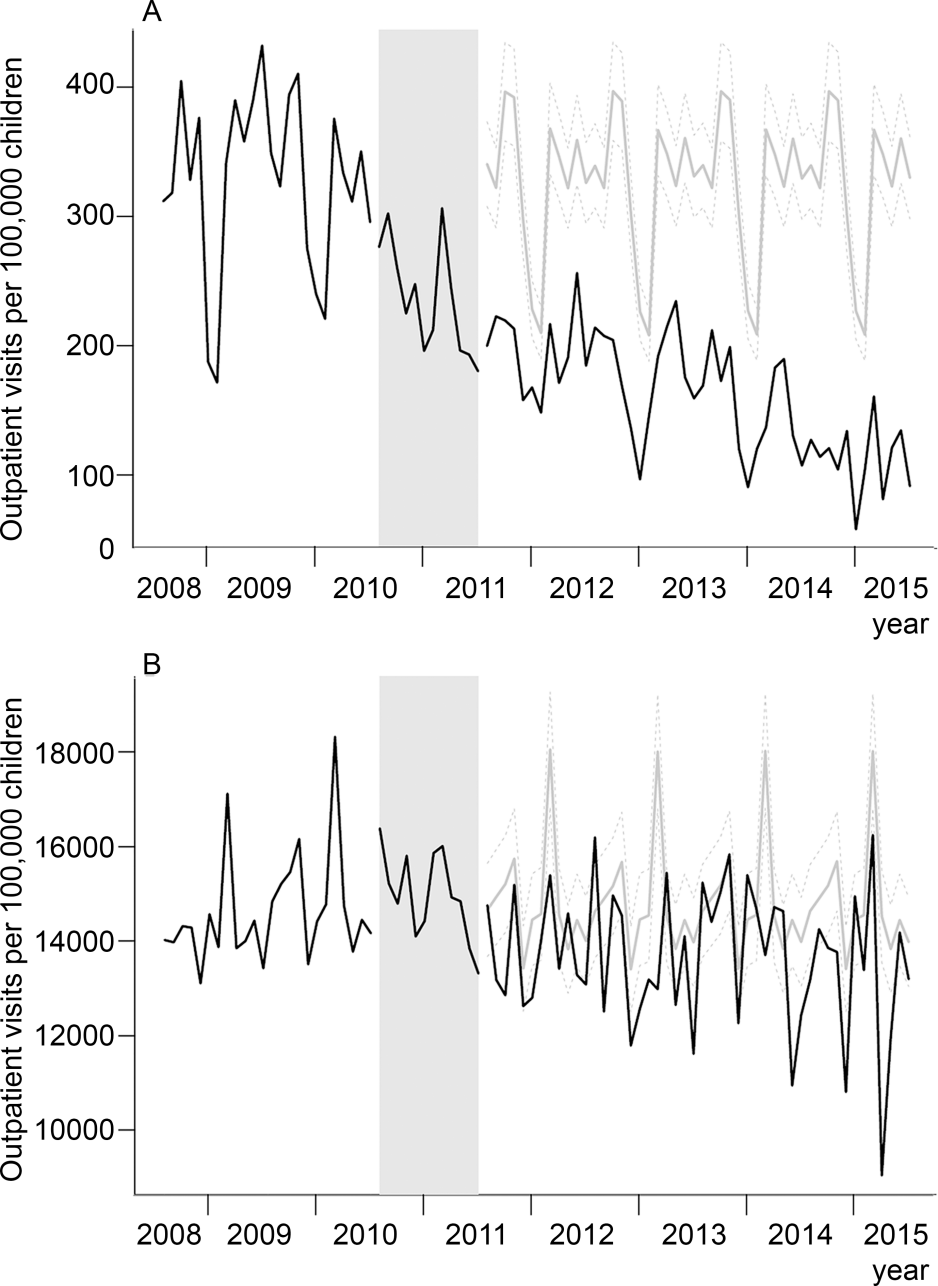
Trends in observed (black lines) and predicted (gray lines) OM (Panel A), and all-cause otitis (Panel B) outpatient visits monthly rates per 100,000 children. Dashed gray lines represent the 95%CI for the predicted rates in the post-vaccination period. The gray bar represents the transition period (August 2010 to July 2011), which was excluded from the time-series analysis.

Average monthly rates of all-cause OM and outpatient visits due to all-other causes for the study period are presented in Table 1. Rates of OM clearly declined especially in 2010–2011 and onwards. Trends in observed and predicted rates of all-cause OM and outpatient visits due to all-other causes among children aged 2 to 23 months are displayed in Fig 1. After PCV10 introduction, there was a downward trend in rates for all-cause OM visits. Observed rates of outpatient visits due to all-other causes also decreased, however the 95% CI of the observed and predicted rates overlapped. In the post-vaccination period, the observed all-cause OM rates in children aged 2–23 months were significantly lower when compared to predicted rates (Table 2). PCV10 vaccination was associated with significant reduction of 50.7% (95% CI 42.2–59.2; p<0.001) in rates of all-cause OM visits. In contrast, reduction in rates for outpatient visits due to all-other causes was non-significant, with a decline of 7.7% (95% CI 0.8–14.7; p<0.001). The resulting estimated impact of PCV10 on all-cause OM was thus 43.0% (95% CI 41.4–44.5; p<0.01) (Table 2).

**Table 1:** Population, case-counts and rates for all-cause OM outpatient visits and all-other causes in children aged 2 to 23 months. Goiânia, Brazil, August 2008-July 2015.

	2008	2009	2010	2011	2012	2013	2014	2015
Population^a^	31,392.46	31,222.79	31,054.04	30,886.21	30,719.28	30,552.88	30,383.76	30,215.27
All-cause OM								
Number of outpatient visits	546	1,258	1,071	789	700	643	478	230
Average of monthly rates^b^^,^^c^	348.50	335.89	287.45	212.90	189.93	175.43	131.12	108.65
All-other causes^a^								
Number of outpatient visits	21,857	55,113	56,217	53,097	51,199	50,529	49,362	28,138
Average of monthly rates^b^^,^^c^	13,950.04	14,713.02	15,089.01	14,328.11	13,891.88	13,785.63	13,540.39	13,290.69

^a^Population estimate, June of each year.

^b^Mean of monthly rates for each year

^c^ Rates per 100,000 population

**Table 2:** Predicted and observed monthly rates for all-cause OM and for all-other causes for children aged 2 to 23 months in the post-vaccination period. Interrupted time-series analysis. Goiânia, August 2011-July 2015.

Outcome	Observed cumulative rates	Predicted cumulative rates	Relative reduction	
			% (95% CI)	P-value
All-cause OM	7,736.78	15,680.33	50.7 (42.2 to 59.2)	<0.001
All-other causes	656,301.62	711,106.89	7.7 (0.8 to 14.7)	<0.001
Difference*			43.0 (41.4 to 44.5)	<0.010

* Relative reduction difference

## Discussion

In this study, we showed a significant impact of PCV10 vaccination on rates of outpatient visits due to all-cause OM in children less than 2 years of age, five years after PCV10 introduction in Brazil. To the best of our knowledge, this is the first population-based study to assess the impact of PCV10 in OM in a developing country, using interrupted time-series analysis. The main advantage of this approach to assess the impact of public health interventions is the capacity to determine predicted rates in the post-intervention period, considering observed rates in the pre-vaccination period. This approach simulates the scenario that would be expected had the intervention not been introduced, in which the secular trends and seasonal variations are taken into account [2, 41, 42]. Our trend analysis showed a seasonal variation on the rates of OM outpatient visits, peaking in the winter (mid-year) and reaching low levels in summer (and school holidays). This seasonal pattern is consistent with the one demonstrated by previous studies on pneumonia hospitalization in children in Goiânia [43].

Selected studies have assessed the impact of PCV on either AOM [17, 18, 44, 45] or OM [2, 14–16, 46–49] outcomes, some of them by means of time-series analysis, with all except one [48] having been conducted in developed countries (S1 and S2 Tables). A study conducted in Canada 3 years after PCV7 introduction in Quebec (2+1 schedule) used administrative data of inpatients and outpatients from the Health Insurance Board and reported a 13.2% reduction in OM rates in children with less than 5 years of age [49]. Another study in UK conducted in children less than 2 years of age used outpatient data from the Intercontinental Medical Statistics and found a reduction of 19.8% in OM rates 4 years after PCV7 introduction (2+1 schedule), and a further 6.6% reduction in OM rates 2 years after PCV13 introduction (2+1 schedule) [46]. Similar results were found in a recent study in Peru in children with less than 1 year of age. The authors reported an overall reduction of 26.0% (95% CI 17.0–34.0%) in AOM rates after PCV introduction (PCV7 from 2009–2011, then replaced by PCV10 from 2012–2013) in a 2+1 schedule [48].

Studies using secondary databases considering ICD codes specific to AOM (S1 table), in general presented higher estimates of impact when compared to those in which OM (S2 table) was considered (thus including ICD-10 code H65 or its equivalent in the ICD-9) [17, 18, 44, 45], ranging from a lower 26% (95% CI 17.0%-34.0%) in Iceland [18] to a high 35% in Italy [45]. Several factors such as age-group considered in the analysis, vaccine coverage and schedule, time after vaccine introduction, and study design, among others, should be considered when interpreting estimates generated by impact studies. These same reasons affect the comparison of different impact estimates. Although selected studies have assessed children younger than 2 years of age [14, 15, 46–48], two studies assessed the impact of PCV7 in children under 5 years of age [2, 16, 49]. In Canada, Wals reported a 13.2% reduction in OM 3 years after PCV introduction [49], while Singleton in the USA reported a 35.5% reduction 5 years after PCV introduction [16].

Most studies evaluating the impact of PCV vaccination on OM were conducted in developed regions and were before-after studies in which the percent difference of OM rates in the pre- and post-vaccination periods were calculated, without adjusting for secular trends and seasonality [2, 14, 17, 45, 47]. The first evidence on PCV impact on OM was generated by a study in the US assessing PCV7 [50]. One year after PCV7 introduction into the US routine childhood immunization, OM outpatient and hospitalized cases in children under 2 years of age decreased by 17% and 28% in Tennessee and upper New York State, respectively [47]. At the national level, in the US, using different data sources, the impact of PCV7 on OM outpatient and inpatient in children under 2 years of age has been estimated as ranging from 20.0% to 42.7%, after 2 and 4 years post-vaccination, respectively [14, 17].

Very few studies have reported the post-vaccination effect of PCV10 alone on OM in infants; none of them applied time-series analysis. In Iceland, the number of AOM outpatient visits and hospitalization in children aged 1–2 years decreased by 26.0% (95% CI 17.0%-34.0%) two after years of PCV10 introduction. In other age groups, no significant change was noted [18]. Two additional studies, conducted in Aboriginal Australian communities [28], and in one emergency department in Northeastern Brazil [29] were not designed to allow for PCV10 impact assessment. The only time-series analysis, which have assessed the effect of PCV10 on OM was conducted in Peru, where PCV7 was introduced in 2009 and later replaced in 2011 for PCV10 [48].

In the present investigation we found a high impact (43.0%; 95% CI 41.4–44.5%) of PCV10 in OM rates. It has been hypothesized that nontypeable *Haemophilus influenzae* (NTHi) protein D, to which PCV10 is conjugated, may play a role in providing further protection against OM caused by either *S*. *pneumoniae* or NTHi [51]. In addition, several may be the factors underlying the high impact of PCV10 on OM outpatient visits. First, our analysis focused on children aged less than 2 years, where disease burden and the expected vaccine impact is higher. Second, our study considered a period of 5 years after PCV introduction. Another factor may be associated to the PCV schedule used in Brazil. In contrast to other countries using 2+1 schedule in which PCV impact in OM have been reported, Brazil introduced PCV10 in a 3+1 schedule. Recently, the NIP has changed from PCV10 schedule from 3+1 to 2+1 based upon a substantial and growing body of research evidence showing that both schedules are protective against carriage, non-invasive and invasive pneumococcal disease [52, 53]. Whether and to what extend the schedule used play a role in the observed impact in Brazil is still to be demonstrated, as no other country have introduced such schedule. Lastly, it is important to consider coding practices by physicians, which may be associated to biases and artifacts in the database. As pointed out by Taylor et al. the choice of the diagnosis codes considered in the analysis can also significantly influence their effectiveness estimates [13]. As studies using secondary data sources, otitis cases are identified according to broad non-specific diagnostic codes that are based on the physician’s clinical assessment. As also suggested by Taylor, physicians may tend to selected the first-listed codes for otitis (H65 in ICD-10) when filling out the diagnosis information in the system, possibly explaining larger decrease estimates, as demonstrated by Zhou et al. [17] and also in our study. This is corroborated by the fact that, when considering different codes reported by year in our database, H65 represent almost 50% of the recorded episodes of all cause otitis media each year. Also, very few cases are coded with 4 digit ICD-10 codes, being 3-digit codes used in the vast majority of cases. Of note, we do not believe that important changes in diagnostic criteria or disease coding practices have occurred in the municipality over the study period.

It is well known that the colonization of nasopharynx by *S*. *pneumoniae* is considered the first-step for development of OM [54]. In a nasopharyngeal carriage survey conducted in children aged 7 to 18 months in Goiânia, we found a significant reduction in colonization by PCV10 serotypes (44%), eight months after PCV10 introduction [25]. Such findings are in accordance with the reduction in outpatient visits due to OM found in the present investigation. Further, these results corroborate recent evidence showing the relationship between vaccine-type pneumococcal carriage and vaccine-type OM [55].

We observed a non-significant decrease in rates of visits due to all-other causes in the post-vaccination period (7.7%; 95% CI 0.8–14.7). A possible explanation for this finding may be the increase in coverage of government social programs such as *Bolsa Família* and Family Health Strategy during the study period, already documented by selected studies [33, 56]. Families receiving the benefit of *Bolsa Familia* cash transfer program are required to routinely access healthcare services for preventive services [33]. Further, several studies have shown that the Family Health Strategy has resulted in improvements in children’s health contributing to the reduction of the most prevalent childhood infectious diseases as diarrhea and respiratory infections [57, 58].

The current study has some limitations. First, the study population represented patients from public primary healthcare facilities and as such did not include the estimated 37.5% of the population using private healthcare system in the municipality, which are likely to be of higher socio-economic status. However, there is no reason to believe that PCV10 impact in children using the private healthcare system should be different. Second, emergency care department visits were not included in the study, since OVIS database did not record such information until 2010, when, despite being incorporated to the system, coverage of emergency rooms and data completeness are reported to be poor. Although emergency rooms were not included in the study, children who arrive without pre-scheduled visits at health centers with symptoms of otitis are seen by the attending physician on the same day, and this service-seeking behavior pattern has not changed after PCV introduction. The lack of information on cases reported from emergency rooms can be responsible for OM burden found in our study lower than the one reported by other studies [2, 14, 15, 17]. However, we do not believe this would significantly affect our impact estimates. So, it is difficult to estimate the extent to which the primary care setting data alone impacted the otitis media prevalence. Third, it is possible that cases of chronic otitis are detected during routine wellness checks at the primary health unit, but to what extent these cases are significant we cannot know. Even though, we believe that the pediatrician who treats this child will most likely record the reason for the consultation as OM, even to justify the prescription of medicine. Fourth, known risk factors for AOM, such as the number of children per household, socioeconomic status, and daycare attendance [59] are not recorded in the OVIS database, and were not considered in our analysis. According to the Brazilian National Household Sample Survey conducted in 2016, the number of day-care attendance in Goiânia has increased since 2010 [60], which could result in increasing risk of pneumococcal transmission among attendees, and hence their young siblings. Finally, generalization of our finding to the country as a whole is not straightforward considering disease burden, demographic, and socio-economic variations among Brazilian regions.

In conclusion, this population-based study provided evidence of the reduction of OM outpatient visits in primary care in children less than 2 years after 5 years of PCV10 introduction into the routine immunization program of Brazil with a 3+1 schedule.

## Supporting information

S1 Dataset Full deidentified dataset in microsoft excel format. (XLSX)

S1 Table Impact of PCV on acute otitis media-related outpatient visits and hospitalizations among children in different regions. (DOCX)

S2 Table Impact of PCV on otitis media-related outpatient visits and hospitalizations among children in different regions. (DOCX)
