# Supplementary material for: Reduction in all-cause otitis media-related outpatient visits in children after PCV10 introduction in Brazil
Source: PLoS One. 2017 Jun 8;12(6):e0179222. doi: 10.1371/journal.pone.0179222 (PMC5464612; doi:10.1371/journal.pone.0179222)
Supplement: S1 Table — (DOCX) [file pone.0179222.s002.docx]

Supporting information

S1 Table. Impact of PCV on acute otitis media-related outpatient visits and hospitalizations among children in different regions.

| **Author,**  **year** | **Services** | **Local** | **Case definition based on ICD-9 Diagnostic Codes** |  | **Case definition based on corresponding ICD-10 codes^a^ and health condition** | **PCV type (schedule)/**  **Time of PCV vaccination** | **Age,**  **years** | **% of reduction**  **(95% CI)** |
| --- | --- | --- | --- | --- | --- | --- | --- | --- |
| Zhou et al., 2008 [[1](#_ENREF_1)] | **Outpatient visits** | US | 381.00 | H65.199 | Other acute nonsuppurative otitis media, unspecified ear | PCV7 (3+1)/ 4 years | <2 | 42.7 (42.4-43.1) |
|  |  |  | 381.01 | H65.00 | Acute serous otitis media, unspecified ear |  |  |  |
|  |  |  | 381.02 | H65.119 | Acute and subacute allergic otitis media (mucoid) (sanguinous) (serous), unspecified ear |  |  |  |
|  |  |  | 381.03 | H65.119 | Acute and subacute allergic otitis media (mucoid) (sanguinous) (serous), unspecified ear |  |  |  |
|  |  |  | 381.04 | H65.119 | Acute and subacute allergic otitis media (mucoid) (sanguinous) (serous), unspecified ear |  |  |  |
|  |  |  | 381.05 | H65.119 | Acute and subacute allergic otitis media (mucoid) (sanguinous) (serous), unspecified ear |  |  |  |
|  |  |  | 381.06 | H65.119 | Acute and subacute allergic otitis media (mucoid) (sanguinous) (serous), unspecified ear |  |  |  |
|  |  |  | 381.4 | H65.90 | Unspecified nonsuppurative otitis media, unspecified ear |  |  |  |
|  |  |  | 382.00 | H66.009 | Acute suppurative otitis media without spontaneous rupture of ear drum, unspecified ear |  |  |  |
|  |  |  | 382.01 | H66.019 | Acute suppurative otitis media with spontaneous rupture of ear drum, unspecified ear |  |  |  |
|  |  |  | 382.02 | H67.9 | Otitis media in diseases classified elsewhere, unspecified ear |  |  |  |
|  |  |  | 382.3 | H66.3X9 | Other chronic suppurative otitis media, unspecified ear |  |  |  |
|  |  |  | 382.9 | H66.90 | Otitis media, unspecified, unspecified ear |  |  |  |
| Magnus et al., 2012 [[2](#_ENREF_2)] | **Outpatient visits** | Norway | Children's disease experienced reported by mothers |  |  | PCV7 (2+1)/ 2 years | <3 | 14.0 (9.0-19) |
| Stamboulidis et al., 2011 [[3](#_ENREF_3)] | **Outpatient visits** | Athens, Greece | Children who were diagnosed with spontaneous draining AOM |  |  | PCV7 (3+1)/ 4 years | <14 | 38.0 |
| Sigurdsson et al., 2015 [[4](#_ENREF_4)] | **Outpatient visits and hospitals** | Iceland |  | H66 | Suppurative and unspecified otitis media | PCV10 (2+1)/3 years | 1-<2 | 26.0 (17.0-34.0) |
|  |  |  |  | H66.0 | Acute suppurative otitis media |  |  |  |
|  |  |  |  | H66.00 | Acute suppurative otitis media without spontaneous rupture of ear drum |  |  |  |
|  |  |  |  | H66.001 | Acute suppurative otitis media without spontaneous rupture of ear drum, right ear |  |  |  |
|  |  |  |  | H66.002 | Acute suppurative otitis media without spontaneous rupture of ear drum, left ear |  |  |  |
|  |  |  |  | H66.003 | Acute suppurative otitis media without spontaneous rupture of ear drum, bilateral |  |  |  |
|  |  |  |  | H66.004 | Acute suppurative otitis media without spontaneous rupture of ear drum, recurrent, right ear |  |  |  |
|  |  |  |  | H66.005 | Acute suppurative otitis media without spontaneous rupture of ear drum, recurrent, left ear |  |  |  |
|  |  |  |  | H66.006 | Acute suppurative otitis media without spontaneous rupture of ear drum, recurrent, bilateral |  |  |  |
|  |  |  |  | H66.007 | Acute suppurative otitis media without spontaneous rupture of ear drum, recurrent, unspecified ear |  |  |  |
|  |  |  |  | H66.009 | Acute suppurative otitis media without spontaneous rupture of ear drum, unspecified ear |  |  |  |
|  |  |  |  | H66.01 | Acute suppurative otitis media with spontaneous rupture of ear drum |  |  |  |
|  |  |  |  | H66.011 | Acute suppurative otitis media with spontaneous rupture of ear drum, right ear |  |  |  |
|  |  |  |  | H66.012 | Acute suppurative otitis media with spontaneous rupture of ear drum, left ear |  |  |  |
|  |  |  |  | H66.013 | Acute suppurative otitis media with spontaneous rupture of ear drum, bilateral |  |  |  |
|  |  |  |  | H66.014 | Acute suppurative otitis media with spontaneous rupture of ear drum, recurrent, right ear |  |  |  |
|  |  |  |  | H66.015 | Acute suppurative otitis media with spontaneous rupture of ear drum recurrent, left ear |  |  |  |
|  |  |  |  | H66.016 | Acute suppurative otitis media with spontaneous rupture of ear drum recurrent, bilateral |  |  |  |
|  |  |  |  | H66.017 | Acute suppurative otitis media with spontaneous rupture of ear drum recurrent, unspecified ear |  |  |  |
|  |  |  |  | H66.019 | Acute suppurative otitis media with spontaneous rupture of ear drum, unspecified ear |  |  |  |
|  |  |  |  | H66.1 | Chronic tubotympanic suppurative otitis media |  |  |  |
|  |  |  |  | H66.10 | Chronic tubotympanic suppurative otitis media. unspecified |  |  |  |
|  |  |  |  | H66.11 | Chronic tubotympanic suppurative otitis media, right ear |  |  |  |
|  |  |  |  | H66.12 | Chronic tubotympanic suppurative otitis media, left ear |  |  |  |
|  |  |  |  | H66.13 | Chronic tubotympanic suppurative otitis media, bilateral |  |  |  |
|  |  |  |  | H66.2 | Chronic atticoantral suppurative otitis media |  |  |  |
|  |  |  |  | H66.20 | Chronic atticoantral suppurative otitis media, unspecified ear |  |  |  |
|  |  |  |  | H66.21 | Chronic atticoantral suppurative otitis media, right ear |  |  |  |
|  |  |  |  | H66.22 | Chronic atticoantral suppurative otitis media, left ear |  |  |  |
|  |  |  |  | H66.23 | Chronic atticoantral suppurative otitis media, bilateral |  |  |  |
|  |  |  |  | H66.3 | Other chronic suppurative otitis media |  |  |  |
|  |  |  |  | H66.3X | Other chronic suppurative otitis media |  |  |  |
|  |  |  |  | H66.3X1 | Other chronic suppurative otitis media, right ear |  |  |  |
|  |  |  |  | H66.3X2 | Other chronic suppurative otitis media, left ear |  |  |  |
|  |  |  |  | H66.3X3 | Other chronic suppurative otitis media, bilateral |  |  |  |
|  |  |  |  | H66.3X9 | Other chronic suppurative otitis mediam, unspecified ear |  |  |  |
|  |  |  |  | H66.4 | Suppurative otitis media, unspecified |  |  |  |
|  |  |  |  | H66.40 | Other chronic suppurative otitis media, unspecified ear |  |  |  |
|  |  |  |  | H66.41 | Other chronic suppurative otitis media right ear |  |  |  |
|  |  |  |  | H66.42 | Other chronic suppurative otitis media left ear |  |  |  |
|  |  |  |  | H66.43 | Other chronic suppurative otitis media bilateral |  |  |  |
|  |  |  |  | H66.9 | Otitis media, unspecified |  |  |  |
|  |  |  |  | H66.90 | Otitis media, unspecified ear |  |  |  |
|  |  |  |  | H66.91 | Otitis media, right ear |  |  |  |
|  |  |  |  | H66.92 | Otitis media, left ear |  |  |  |
|  |  |  |  | H66.93 | Otitis media, bilateral |  |  |  |
| Ansaldi et al., 2008 [[5](#_ENREF_5)] | **Hospitals** | administrative region of Liguria, Italy | 382.00 | H66.009 | Acute suppurative otitis media without spontaneous rupture of ear drum, unspecified ear | PCV7 (2+1)/ 2 years | <2 | 36.4 (24.1-46.7) |
|  |  |  | 381.01 | H66.019 | Acute suppurative otitis media with spontaneous rupture of ear drum, unspecified ear |  |  |  |
|  |  |  | 382.02 | H67.9 | Otitis media in diseases classified elsewhere, unspecified ear |  |  |  |
|  |  |  | 382.1 | H66.13 | Chronic tubotympanic suppurative otitis media, bilateral |  |  |  |
|  |  |  | 382.2 | H66.23 | Chronic atticoantral suppurative otitis media, bilateral |  |  |  |
|  |  |  | 382.3 | H66.3X9 | Other chronic suppurative otitis media, unspecified ear |  |  |  |
|  |  |  | 382.4 | H66.40 | Suppurative otitis media, unspecified, unspecified ear |  |  |  |
|  |  |  | 382.9 | H66.90 | Otitis media, unspecified, unspecified ear |  |  |  |
| Fortunato et al., 2015 [[6](#_ENREF_6)] | **Hospitals** | Italy | 382.00 | H66.009 | Acute suppurative otitis media without spontaneous rupture of ear drum, unspecified ear | PCV7 (2+1)/ 4 years | <5 | 39.0 (35.0-42.0) |
|  |  |  | 382.01 | H66.019 | Acute suppurative otitis media with spontaneous rupture of ear drum, unspecified ear | PCV13 (2+1)/ 1 year |  |  |
|  |  |  | 382.02 | H67.9 | Otitis media in diseases classified elsewhere, unspecified ear |  |  |  |
|  |  |  | 382.1 | H66.13 | Chronic tubotympanic suppurative otitis media, bilateral |  |  |  |
|  |  |  | 382.2 | H66.23 | Chronic atticoantral suppurative otitis media, bilateral |  |  |  |
|  |  |  | 382.3 | H66.3X9 | Other chronic suppurative otitis media, unspecified ear |  |  |  |
|  |  |  | 382.4 | H66.40 | Suppurative otitis media, unspecified, unspecified ear |  |  |  |
|  |  |  | 382.9 | H66.90 | Otitis media, unspecified, unspecified ear |  |  |  |

^a^ ICD-10 it is not completely equivalent to the source ICD-9.

AOM: Acute Otitis Media; CI: Confidence Interval; ICD: International Classification of Diseases (9th Revision and 10th Revision); NOS: Not Otherwise Specified; OM: Otitis Media; PCV: Pneumococcal Conjugate Vaccine (7, 10 and 13-valent); TM: Tympanic membrane; TMP: Tympanic membrane perforation; US: United States

**References**

1. Zhou F, Shefer A, Kong Y, Nuorti JP. Trends in acute otitis media-related health care utilization by privately insured young children in the United States, 1997-2004. Pediatrics. 2008;121(2):253-60. doi: 10.1542/peds.2007-0619. PubMed PMID: 18245415.

2. Magnus MC, Vestrheim DF, Nystad W, Haberg SE, Stigum H, London SJ, et al. Decline in early childhood respiratory tract infections in the Norwegian mother and child cohort study after introduction of pneumococcal conjugate vaccination. Pediatr Infect Dis J. 2012;31(9):951-5. doi: 10.1097/INF.0b013e31825d2f76. PubMed PMID: 22627867; PubMed Central PMCID: PMC3421039.

3. Stamboulidis K, Chatzaki D, Poulakou G, Ioannidou S, Lebessi E, Katsarolis I, et al. The impact of the heptavalent pneumococcal conjugate vaccine on the epidemiology of acute otitis media complicated by otorrhea. Pediatr Infect Dis J. 2011;30(7):551-5. doi: 10.1097/INF.0b013e31821038d9. PubMed PMID: 21297521.

4. Sigurdsson S, Kristinsson KG, Erlendsdottir H, Hrafnkelsson B, Haraldsson A. Decreased Incidence of Respiratory Infections in Children After Vaccination with Ten-valent Pneumococcal Vaccine. Pediatr Infect Dis J. 2015;34(12):1385-90. doi: 10.1097/INF.0000000000000899. PubMed PMID: 26780024.

5. Ansaldi F, Sticchi L, Durando P, Carloni R, Oreste P, Vercelli M, et al. Decline in pneumonia and acute otitis media after the introduction of childhood pneumococcal vaccination in Liguria, Italy. J Int Med Res. 2008;36(6):1255-60. doi: 10.1177/147323000803600612. PubMed PMID: 19094434.

6. Fortunato F, Martinelli D, Cappelli MG, Cozza V, Prato R. Impact of Pneumococcal Conjugate Universal Routine Vaccination on Pneumococcal Disease in Italian Children. J Immunol Res. 2015;2015:206757. doi: 10.1155/2015/206757. PubMed PMID: 26351644; PubMed Central PMCID: PMC4553180.
