# Supplementary material for: Reduction in all-cause otitis media-related outpatient visits in children after PCV10 introduction in Brazil
Source: PLoS One. 2017 Jun 8;12(6):e0179222. doi: 10.1371/journal.pone.0179222 (PMC5464612; doi:10.1371/journal.pone.0179222)
Supplement: S2 Table — (DOCX) [file pone.0179222.s003.docx]

Supporting information

S2 Table. Impact of PCV on otitis media-related outpatient visits and hospitalizations among children in different regions.

| **Services** | **Author,**  **year** | **Locality** | **Case definition based on ICD-9 Diagnostic Codes** | **Case definition based on corresponding ICD-10 codes^a^ and health condition** | | **PCV type (schedule)/**  **Time of PCV vaccination** | **Age,**  **years** | **% of reduction**  **(95% CI)** |
| --- | --- | --- | --- | --- | --- | --- | --- | --- |
| Outpatient visits and hospitals | Grijalva et al., 2006 [[1](#_ENREF_1)] | US | 381.00 | H65.199 | Other acute nonsuppurative otitis media, unspecified ear | PCV7 (3+1)/ 3 years | <2 | 20.0 (4.0-34.0) |
|  |  |  | 381.01 | H65.00 | Acute serous otitis media, unspecified ear |  |  |  |
|  |  |  | 381.02 | H65.119 | Acute and subacute allergic otitis media (mucoid) (sanguinous) (serous), unspecified ear |  |  |  |
|  |  |  | 381.03 | H65.119 | Acute and subacute allergic otitis media (mucoid) (sanguinous) (serous), unspecified ear |  |  |  |
|  |  |  | 381.04 | H65.119 | Acute and subacute allergic otitis media (mucoid) (sanguinous) (serous), unspecified ear |  |  |  |
|  |  |  | 381.05 | H65.119 | Acute and subacute allergic otitis media (mucoid) (sanguinous) (serous), unspecified ear |  |  |  |
|  |  |  | 381.06 | H65.119 | Acute and subacute allergic otitis media (mucoid) (sanguinous) (serous), unspecified ear |  |  |  |
|  |  |  | 381.10 | H65.20 | Chronic serous otitis media, unspecified ear |  |  |  |
|  |  |  | 381.19 | H65.20 | Chronic serous otitis media, unspecified ear |  |  |  |
|  |  |  | 381.20 | H65.30 | Chronic mucoid otitis media, unspecified ear |  |  |  |
|  |  |  | 381.29 | H65.30 | Chronic mucoid otitis media, unspecified ear |  |  |  |
|  |  |  | 381.3 | H65.499 | Other chronic nonsuppurative otitis media, unspecified ear |  |  |  |
|  |  |  | 381.4 | H65.90 | Unspecified nonsuppurative otitis media, unspecified ear |  |  |  |
|  |  |  | 381.50 | H68.009 | Unspecified Eustachian salpingitis, unspecified ear |  |  |  |
|  |  |  | 381.51 | H68.019 | Acute Eustachian salpingitis, unspecified ear |  |  |  |
|  |  |  | 381.52 | H68.029 | Chronic Eustachian salpingitis, unspecified ear |  |  |  |
|  |  |  | 381.60 | H68.109 | Unspecified obstruction of Eustachian tube, unspecified ear |  |  |  |
|  |  |  | 381.61 | H68.119 | Osseous obstruction of Eustachian tube, unspecified ear |  |  |  |
|  |  |  | 381.62 | H68.129 | Intrinsic cartilagenous obstruction of Eustachian tube, unspecified ear |  |  |  |
|  |  |  | 381.63 | H68.139 | Extrinsic cartilagenous obstruction of Eustachian tube, unspecified ear |  |  |  |
|  |  |  | 381.7 | H69.00 | Patulous Eustachian tube, unspecified ear |  |  |  |
|  |  |  | 381.81 | H69.80 | Other specified disorders of Eustachian tube, unspecified ear |  |  |  |
|  |  |  | 381.89 | H69.80 | Other specified disorders of Eustachian tube, unspecified ear |  |  |  |
|  |  |  | 381.9 | H69.90 | Unspecified Eustachian tube disorder, unspecified ear |  |  |  |
|  |  |  | 382.00 | H66.009 | Acute suppurative otitis media without spontaneous rupture of ear drum, unspecified ear |  |  |  |
|  |  |  | 382.01 | H66.019 | Acute suppurative otitis media with spontaneous rupture of ear drum, unspecified ear |  |  |  |
|  |  |  | 382.02 | H67.9 | Otitis media in diseases classified elsewhere, unspecified ear |  |  |  |
|  |  |  | 382.1 | H66.13 | Chronic tubotympanic suppurative otitis media, bilateral |  |  |  |
|  |  |  | 382.2 | H66.23 | Chronic atticoantral suppurative otitis media, bilateral |  |  |  |
|  |  |  | 382.3 | H66.3X9 | Other chronic suppurative otitis media, unspecified ear |  |  |  |
|  |  |  | 382.4 | H66.40 | Suppurative otitis media, unspecified, unspecified ear |  |  |  |
|  |  |  | 382.9 | H66.90 | Otitis media, unspecified, unspecified ear |  |  |  |
| Outpatient visits and hospitals | Poehling et al., 2007 [[2](#_ENREF_2)] | Tennesse and upstate | 381.00 | H65.199 | Other acute nonsuppurative otitis media, unspecified ear | PCV7 (3+1)/ 1 year | <2 | 17.0 (14.0-19.0) in Tennesse |
|  |  |  | 381.01 | H65.00 | Acute serous otitis media, unspecified ear |  |  |  |
|  |  | New York, US | 381.02 | H65.119 | Acute and subacute allergic otitis media (mucoid) (sanguinous) (serous), unspecified ear |  |  | 28.0 (23.0-33.0) in upstate New York |
|  |  |  | 381.03 | H65.119 | Acute and subacute allergic otitis media (mucoid) (sanguinous) (serous), unspecified ear |  |  |  |
|  |  |  | 381.04 | H65.119 | Acute and subacute allergic otitis media (mucoid) (sanguinous) (serous), unspecified ear |  |  |  |
|  |  |  | 381.05 | H65.119 | Acute and subacute allergic otitis media (mucoid) (sanguinous) (serous), unspecified ear |  |  |  |
|  |  |  | 381.06 | H65.119 | Acute and subacute allergic otitis media (mucoid) (sanguinous) (serous), unspecified ear |  |  |  |
|  |  |  | 381.10 | H65.20 | Chronic serous otitis media, unspecified ear |  |  |  |
|  |  |  | 381.19 | H65.20 | Chronic serous otitis media, unspecified ear |  |  |  |
|  |  |  | 381.20 | H65.30 | Chronic mucoid otitis media, unspecified ear |  |  |  |
|  |  |  | 381.29 | H65.30 | Chronic mucoid otitis media, unspecified ear |  |  |  |
|  |  |  | 381.3 | H65.499 | Other chronic nonsuppurative otitis media, unspecified ear |  |  |  |
|  |  |  | 381.4 | H65.90 | Unspecified nonsuppurative otitis media, unspecified ear |  |  |  |
|  |  |  | 382.00 | H66.009 | Acute suppurative otitis media without spontaneous rupture of ear drum, unspecified ear |  |  |  |
|  |  |  | 382.01 | H66.019 | Acute suppurative otitis media with spontaneous rupture of ear drum, unspecified ear |  |  |  |
|  |  |  | 382.02 | H67.9 | Otitis media in diseases classified elsewhere, unspecified ear |  |  |  |
|  |  |  | 382.1 | H66.13 | Chronic tubotympanic suppurative otitis media, bilateral |  |  |  |
|  |  |  | 382.2 | H66.23 | Chronic atticoantral suppurative otitis media, bilateral |  |  |  |
|  |  |  | 382.3 | H66.3X9 | Other chronic suppurative otitis media, unspecified ear |  |  |  |
|  |  |  | 382.4 | H66.40 | Suppurative otitis media, unspecified, unspecified ear |  |  |  |
|  |  |  | 382.9 | H66.90 | Otitis media, unspecified, unspecified ear |  |  |  |
| Outpatient visits and hospitals | Grijalva et al., 2009 [[3](#_ENREF_3)] | US | 381.00 | H65.199 | Other acute nonsuppurative otitis media, unspecified ear | PCV7 (3+1)/ 6 years | <5 | 33.0 (22.0-43.0) |
|  |  |  | 381.01 | H65.00 | Acute serous otitis media, unspecified ear |  |  |  |
|  |  |  | 381.02 | H65.119 | Acute and subacute allergic otitis media (mucoid) (sanguinous) (serous), unspecified ear |  |  |  |
|  |  |  | 381.03 | H65.119 | Acute and subacute allergic otitis media (mucoid) (sanguinous) (serous), unspecified ear |  |  |  |
|  |  |  | 381.04 | H65.119 | Acute and subacute allergic otitis media (mucoid) (sanguinous) (serous), unspecified ear |  |  |  |
|  |  |  | 381.05 | H65.119 | Acute and subacute allergic otitis media (mucoid) (sanguinous) (serous), unspecified ear |  |  |  |
|  |  |  | 381.06 | H65.119 | Acute and subacute allergic otitis media (mucoid) (sanguinous) (serous), unspecified ear |  |  |  |
|  |  |  | 381.10 | H65.20 | Chronic serous otitis media, unspecified ear |  |  |  |
|  |  |  | 381.19 | H65.20 | Chronic serous otitis media, unspecified ear |  |  |  |
|  |  |  | 381.20 | H65.30 | Chronic mucoid otitis media, unspecified ear |  |  |  |
|  |  |  | 381.29 | H65.30 | Chronic mucoid otitis media, unspecified ear |  |  |  |
|  |  |  | 381.3 | H65.499 | Other chronic nonsuppurative otitis media, unspecified ear |  |  |  |
|  |  |  | 381.4 | H65.90 | Unspecified nonsuppurative otitis media, unspecified ear |  |  |  |
|  |  |  | 381.50 | H68.009 | Unspecified Eustachian salpingitis, unspecified ear |  |  |  |
|  |  |  | 381.51 | H68.019 | Acute Eustachian salpingitis, unspecified ear |  |  |  |
|  |  |  | 381.52 | H68.029 | Chronic Eustachian salpingitis, unspecified ear |  |  |  |
|  |  |  | 381.60 | H68.109 | Unspecified obstruction of Eustachian tube, unspecified ear |  |  |  |
|  |  |  | 381.61 | H68.119 | Osseous obstruction of Eustachian tube, unspecified ear |  |  |  |
|  |  |  | 381.62 | H68.129 | Intrinsic cartilagenous obstruction of Eustachian tube, unspecified ear |  |  |  |
|  |  |  | 381.63 | H68.139 | Extrinsic cartilagenous obstruction of Eustachian tube, unspecified ear |  |  |  |
|  |  |  | 381.7 | H69.00 | Patulous Eustachian tube, unspecified ear |  |  |  |
|  |  |  | 381.81 | H69.80 | Other specified disorders of Eustachian tube, unspecified ear |  |  |  |
|  |  |  | 381.89 | H69.80 | Other specified disorders of Eustachian tube, unspecified ear |  |  |  |
|  |  |  | 381.9 | H69.90 | Unspecified Eustachian tube disorder, unspecified ear |  |  |  |
|  |  |  | 382.00 | H66.009 | Acute suppurative otitis media without spontaneous rupture of ear drum, unspecified ear |  |  |  |
|  |  |  | 382.01 | H66.019 | Acute suppurative otitis media with spontaneous rupture of ear drum, unspecified ear |  |  |  |
|  |  |  | 382.02 | H67.9 | Otitis media in diseases classified elsewhere, unspecified ear |  |  |  |
|  |  |  | 382.1 | H66.13 | Chronic tubotympanic suppurative otitis media, bilateral |  |  |  |
|  |  |  | 382.2 | H66.23 | Chronic atticoantral suppurative otitis media, bilateral |  |  |  |
|  |  |  | 382.3 | H66.3X9 | Other chronic suppurative otitis media, unspecified ear |  |  |  |
|  |  |  | 382.4 | H66.40 | Suppurative otitis media, unspecified, unspecified ear |  |  |  |
|  |  |  | 382.9 | H66.90 | Otitis media, unspecified, unspecified ear |  |  |  |
| Outpatient visits and hospitals | Singleton et al., 2009 [[4](#_ENREF_4)] | US | 381.00 | H65.199 | Other acute nonsuppurative otitis media, unspecified ear | PCV7 (3+1)/ 5 years | <5 | 35.5 |
|  |  |  | 381.01 | H65.00 | Acute serous otitis media, unspecified ear |  |  |  |
|  |  |  | 381.02 | H65.119 | Acute and subacute allergic otitis media (mucoid) (sanguinous) (serous), unspecified ear |  |  |  |
|  |  |  | 381.03 | H65.119 | Acute and subacute allergic otitis media (mucoid) (sanguinous) (serous), unspecified ear |  |  |  |
|  |  |  | 381.04 | H65.119 | Acute and subacute allergic otitis media (mucoid) (sanguinous) (serous), unspecified ear |  |  |  |
|  |  |  | 381.05 | H65.119 | Acute and subacute allergic otitis media (mucoid) (sanguinous) (serous), unspecified ear |  |  |  |
|  |  |  | 381.06 | H65.119 | Acute and subacute allergic otitis media (mucoid) (sanguinous) (serous), unspecified ear |  |  |  |
|  |  |  | 381.10 | H65.20 | Chronic serous otitis media, unspecified ear |  |  |  |
|  |  |  | 381.19 | H65.20 | Chronic serous otitis media, unspecified ear |  |  |  |
|  |  |  | 381.20 | H65.30 | Chronic mucoid otitis media, unspecified ear |  |  |  |
|  |  |  | 381.29 | H65.30 | Chronic mucoid otitis media, unspecified ear |  |  |  |
|  |  |  | 381.3 | H65.499 | Other chronic nonsuppurative otitis media, unspecified ear |  |  |  |
|  |  |  | 381.4 | H65.90 | Unspecified nonsuppurative otitis media, unspecified ear |  |  |  |
|  |  |  | 382.00 | H66.009 | Acute suppurative otitis media without spontaneous rupture of ear drum, unspecified ear |  |  |  |
|  |  |  | 381.01 | H66.019 | Acute suppurative otitis media with spontaneous rupture of ear drum, unspecified ear |  |  |  |
|  |  |  | 382.02 | H67.9 | Otitis media in diseases classified elsewhere, unspecified ear |  |  |  |
|  |  |  | 382.1 | H66.13 | Chronic tubotympanic suppurative otitis media, bilateral |  |  |  |
|  |  |  | 382.2 | H66.23 | Chronic atticoantral suppurative otitis media, bilateral |  |  |  |
|  |  |  | 382.3 | H66.3X9 | Other chronic suppurative otitis media, unspecified ear |  |  |  |
|  |  |  | 382.4 | H66.40 | Suppurative otitis media, unspecified, unspecified ear |  |  |  |
|  |  |  | 382.9 | H66.90 | Otitis media, unspecified, unspecified ear |  |  |  |
| Outpatient visits and hospitals | Wals et al., 2009 [[5](#_ENREF_5)] | Quebec, Canada | 381.00 | H65.199 | Other acute nonsuppurative otitis media, unspecified ear | PCV7 (2+1)/ 3 years | <5 | 13.2 |
|  |  |  | 381.01 | H65.00 | Acute serous otitis media, unspecified ear |  |  |  |
|  |  |  | 381.02 | H65.119 | Acute and subacute allergic otitis media (mucoid) (sanguinous) (serous), unspecified ear |  |  |  |
|  |  |  | 381.03 | H65.119 | Acute and subacute allergic otitis media (mucoid) (sanguinous) (serous), unspecified ear |  |  |  |
|  |  |  | 381.04 | H65.119 | Acute and subacute allergic otitis media (mucoid) (sanguinous) (serous), unspecified ear |  |  |  |
|  |  |  | 381.05 | H65.119 | Acute and subacute allergic otitis media (mucoid) (sanguinous) (serous), unspecified ear |  |  |  |
|  |  |  | 381.06 | H65.119 | Acute and subacute allergic otitis media (mucoid) (sanguinous) (serous), unspecified ear |  |  |  |
|  |  |  | 381.10 | H65.20 | Chronic serous otitis media, unspecified ear |  |  |  |
|  |  |  | 381.19 | H65.20 | Chronic serous otitis media, unspecified ear |  |  |  |
|  |  |  | 381.20 | H65.30 | Chronic mucoid otitis media, unspecified ear |  |  |  |
|  |  |  | 381.29 | H65.30 | Chronic mucoid otitis media, unspecified ear |  |  |  |
|  |  |  | 381.3 | H65.499 | Other chronic nonsuppurative otitis media, unspecified ear |  |  |  |
|  |  |  | 381.4 | H65.90 | Unspecified nonsuppurative otitis media, unspecified ear |  |  |  |
|  |  |  | 381.50 | H68.009 | Unspecified Eustachian salpingitis, unspecified ear |  |  |  |
|  |  |  | 381.51 | H68.019 | Acute Eustachian salpingitis, unspecified ear |  |  |  |
|  |  |  | 381.52 | H68.029 | Chronic Eustachian salpingitis, unspecified ear |  |  |  |
|  |  |  | 381.60 | H68.109 | Unspecified obstruction of Eustachian tube, unspecified ear |  |  |  |
|  |  |  | 381.61 | H68.119 | Osseous obstruction of Eustachian tube, unspecified ear |  |  |  |
|  |  |  | 381.62 | H68.129 | Intrinsic cartilagenous obstruction of Eustachian tube, unspecified ear |  |  |  |
|  |  |  | 381.63 | H68.139 | Extrinsic cartilagenous obstruction of Eustachian tube, unspecified ear |  |  |  |
|  |  |  | 381.7 | H69.00 | Patulous Eustachian tube, unspecified ear |  |  |  |
|  |  |  | 381.81 | H69.80 | Other specified disorders of Eustachian tube, unspecified ear |  |  |  |
|  |  |  | 381.89 | H69.80 | Other specified disorders of Eustachian tube, unspecified ear |  |  |  |
|  |  |  | 381.9 | H69.90 | Unspecified Eustachian tube disorder, unspecified ear |  |  |  |
|  |  |  | 382.00 | H66.009 | Acute suppurative otitis media without spontaneous rupture of ear drum, unspecified ear |  |  |  |
|  |  |  | 382.01 | H66.019 | Acute suppurative otitis media with spontaneous rupture of ear drum, unspecified ear |  |  |  |
|  |  |  | 382.02 | H67.9 | Otitis media in diseases classified elsewhere, unspecified ear |  |  |  |
|  |  |  | 382.1 | H66.13 | Chronic tubotympanic suppurative otitis media, bilateral |  |  |  |
|  |  |  | 382.2 | H66.23 | Chronic atticoantral suppurative otitis media, bilateral |  |  |  |
|  |  |  | 382.3 | H66.3X9 | Other chronic suppurative otitis media, unspecified ear |  |  |  |
|  |  |  | 382.4 | H66.40 | Suppurative otitis media, unspecified, unspecified ear |  |  |  |
|  |  |  | 382.9 | H66.90 | Otitis media, unspecified, unspecified ear |  |  |  |
| Outpatient visits and hospitals | Marom et al., 2014 [[6](#_ENREF_6)] | US | 381.00 | H65.199 | Other acute nonsuppurative otitis media, unspecified ear | PCV7 (3+1)/ 9 years | <2 | 0.03 child/year |
|  |  |  | 381.01 | H65.00 | Acute serous otitis media, unspecified ear |  |  |  |
|  |  |  | 381.02 | H65.119 | Acute and subacute allergic otitis media (mucoid) (sanguinous) (serous), unspecified ear | PCV13 (3+1)/ 2 years |  | 0.27 child/year |
|  |  |  | 381.03 | H65.119 | Acute and subacute allergic otitis media (mucoid) (sanguinous) (serous), unspecified ear |  |  |  |
|  |  |  | 381.04 | H65.119 | Acute and subacute allergic otitis media (mucoid) (sanguinous) (serous), unspecified ear |  |  |  |
|  |  |  | 381.05 | H65.119 | Acute and subacute allergic otitis media (mucoid) (sanguinous) (serous), unspecified ear |  |  |  |
|  |  |  | 381.06 | H65.119 | Acute and subacute allergic otitis media (mucoid) (sanguinous) (serous), unspecified ear |  |  |  |
|  |  |  | 381.10 | H65.20 | Chronic serous otitis media, unspecified ear |  |  |  |
|  |  |  | 381.19 | H65.20 | Chronic serous otitis media, unspecified ear |  |  |  |
|  |  |  | 381.20 | H65.30 | Chronic mucoid otitis media, unspecified ear |  |  |  |
|  |  |  | 381.29 | H65.30 | Chronic mucoid otitis media, unspecified ear |  |  |  |
|  |  |  | 381.3 | H65.499 | Other chronic nonsuppurative otitis media, unspecified ear |  |  |  |
|  |  |  | 381.4 | H65.90 | Unspecified nonsuppurative otitis media, unspecified ear |  |  |  |
|  |  |  | 381.50 | H68.009 | Unspecified Eustachian salpingitis, unspecified ear |  |  |  |
|  |  |  | 381.51 | H68.019 | Acute Eustachian salpingitis, unspecified ear |  |  |  |
|  |  |  | 381.52 | H68.029 | Chronic Eustachian salpingitis, unspecified ear |  |  |  |
|  |  |  | 381.60 | H68.109 | Unspecified obstruction of Eustachian tube, unspecified ear |  |  |  |
|  |  |  | 381.61 | H68.119 | Osseous obstruction of Eustachian tube, unspecified ear |  |  |  |
|  |  |  | 381.62 | H68.129 | Intrinsic cartilagenous obstruction of Eustachian tube, unspecified ear |  |  |  |
|  |  |  | 381.63 | H68.139 | Extrinsic cartilagenous obstruction of Eustachian tube, unspecified ear |  |  |  |
|  |  |  | 381.7 | H69.00 | Patulous Eustachian tube, unspecified ear |  |  |  |
|  |  |  | 381.81 | H69.80 | Other specified disorders of Eustachian tube, unspecified ear |  |  |  |
|  |  |  | 381.89 | H69.80 | Other specified disorders of Eustachian tube, unspecified ear |  |  |  |
|  |  |  | 381.9 | H69.90 | Unspecified Eustachian tube disorder, unspecified ear |  |  |  |
|  |  |  | 382.00 | H66.009 | Acute suppurative otitis media without spontaneous rupture of ear drum, unspecified ear |  |  |  |
|  |  |  | 382.01 | H66.019 | Acute suppurative otitis media with spontaneous rupture of ear drum, unspecified ear |  |  |  |
|  |  |  | 382.02 | H67.9 | Otitis media in diseases classified elsewhere, unspecified ear |  |  |  |
|  |  |  | 382.1 | H66.13 | Chronic tubotympanic suppurative otitis media, bilateral |  |  |  |
|  |  |  | 382.2 | H66.23 | Chronic atticoantral suppurative otitis media, bilateral |  |  |  |
|  |  |  | 382.3 | H66.3X9 | Other chronic suppurative otitis media, unspecified ear |  |  |  |
|  |  |  | 382.4 | H66.40 | Suppurative otitis media, unspecified, unspecified ear |  |  |  |
|  |  |  | 382.9 | H66.90 | Otitis media, unspecified, unspecified ear |  |  |  |
|  |  |  | 394.00 | H73.009 | Acute myringitis, unspecified ear |  |  |  |
|  |  |  | 384.01 | H73.019 | Bullous myringitis, unspecified ear |  |  |  |
|  |  |  | 384.09 | H73.099 | Other acute myringitis, unspecified ear |  |  |  |
|  |  |  | 384.1 | H73.10 | Chronic myringitis, unspecified ear |  |  |  |
|  |  |  | 384.20 | H72.9 | Unspecified perforation of tympanic membrane, unspecified ear |  |  |  |
|  |  |  | 384.21 | H72.00 | Central perforation of tympanic membrane, unspecified ear |  |  |  |
|  |  |  | 384.22 | H72.10 | Attic perforation of tympanic membrane, unspecified ear |  |  |  |
|  |  |  | 384.23 | H72.2X9 | Other marginal perforations of tympanic membrane, unspecified ear |  |  |  |
|  |  |  | 384.24 | H72.819 | Multiple perforations of tympanic membrane, unspecified ear |  |  |  |
|  |  |  | 384.25 | H72.829 | Total perforations of tympanic membrane, unspecified ear |  |  |  |
|  |  |  | 384.81 | H73.819 | Atrophic flaccid tympanic membrane, unspecified ear |  |  |  |
|  |  |  | 384.82 | H73.829 | Atrophic nonflaccid tympanic membrane, unspecified ear |  |  |  |
|  |  |  | 384.9 | H73.93 | Unspecified disorder of tympanic membrane, bilateral |  |  |  |
| Outpatient visits | Leach et al., 2014 [[7](#_ENREF_7)] | Remote communities in Northern and Western Territory, Australia | Middle ear discharge with or without TMP (Middle ear discharge observed with or without TMP (recently healed or present for less than six weeks or covering less than 2% of the pars tensa) |  |  | PCV7 (3+1) and PCV10 (3+1) )/ 3 years | <3 | 16.0 (8.0-24.0) |
| Outpatient visits and hospitals | Ben-Shimol et al., 2014 [[8](#_ENREF_8)] | Southern Israel | Children who presented acute symptoms (<7 days), including ≥1 of the following: fever, abnormal TM otoscopic ﬁndings (erythema, opacity, bulging, or draining ears) |  |  | PCV7 (3+0)/ 2 years | <2 | 31.0 |
|  |  |  |  |  |  | PCV13 (3+0)/ 2,5 years |  | 46.0^b^ |
| Outpatient visits | Lau et al., 2015 [[9](#_ENREF_9)] | England |  |  | Nonsuppurative otitis media + eustachio | PCV7 (2+1)/ 4 years | <2 | 19.8 (16.0-23.5)^b^ |
|  |  |  |  |  | Acute non suppurative otitis media | PCV13 (2+1)/ 2 years |  | 6.6 (1.9-11.0)^b^ |
|  |  |  |  |  | Suppurative otitis media |  |  |  |
|  |  |  |  |  | Chronic otitis media with effusion-mucoid |  |  |  |
|  |  |  |  |  | Acute nonsuppurative otitis media |  |  |  |
|  |  |  |  |  | Nonsuppurative otitis media |  |  |  |
|  |  |  |  |  | Chronic otitis media with effusion-serous |  |  |  |
|  |  |  |  |  | Chronic suppurative otitis media NOS |  |  |  |
|  |  |  |  |  | Purulent otitis media NOS |  |  |  |
|  |  |  |  |  | Chronic mucoid otitis media |  |  |  |
|  |  |  |  |  | Acute nonsupurative otitis media NOS |  |  |  |
|  |  |  |  |  | Chronic otitis media with effusion-unspecified |  |  |  |
|  |  |  |  |  | Nonsuppurative otitis media unspecified |  |  |  |
|  |  |  |  |  | Serous otitis media NOS |  |  |  |
|  |  |  |  |  | Chronic purulent otitis media |  |  |  |
|  |  |  |  |  | Acute mucoid otitis media |  |  |  |
|  |  |  |  |  | Chronic suppurative otitis media-tubotympanic |  |  |  |
|  |  |  |  |  | Catarrhal otitis media NOS |  |  |  |
|  |  |  |  |  | Chronic otitis media with effusion-other |  |  |  |
|  |  |  |  |  | Chronic mucoid otitis media NOS |  |  |  |
|  |  |  |  |  | Chronic serous otitis media |  |  |  |
|  |  |  |  |  | Bilateral chronic serous otitis |  |  |  |
|  |  |  |  |  | Chronic serous otitis media NOS |  |  |  |
|  |  |  |  |  | Nonsuppurative otitis media NOS |  |  |  |
|  |  |  |  |  | Chronic suppurative otitis media-atticoantral |  |  |  |
|  |  |  |  |  | Mucoid otitis media NOS |  |  |  |
|  |  |  |  |  | Chronic otitis media with effussion-purulent |  |  |  |
|  |  |  |  |  | Acute sanguinous otitis media |  |  |  |
|  |  |  |  |  | Acute allergic mucoid otitis media |  |  |  |
|  |  |  |  |  | Mucosanguinous chronic otitis media |  |  |  |
|  |  |  |  |  | Otitis media NOS |  |  |  |
|  |  |  |  |  | Acute suppurative otitis media |  |  |  |
|  |  |  |  |  | Otitis media NOS |  |  |  |
|  |  |  |  |  | Acute right otitis media |  |  |  |
|  |  |  |  |  | Acute left otitis media |  |  |  |
|  |  |  |  |  | Acute bilateral otitis media |  |  |  |
|  |  |  |  |  | Acute suppurative otitis media |  |  |  |
|  |  |  |  |  | Suppurative otitis media |  |  |  |
|  |  |  |  |  | Acute otitis media with effusion |  |  |  |
|  |  |  |  |  | Acute serous otitis media |  |  |  |
|  |  |  |  |  | Acute suppurative otitis media-drum |  |  |  |
|  |  |  |  |  | Acute suppurative otitis media+drum rupture |  |  |  |
|  |  |  |  |  | Acute suppurative otitis media NOS |  |  |  |
|  |  |  |  |  | Bilateral suppurative otitis media |  |  |  |
|  |  |  |  |  | Postmeasles otitis media |  |  |  |
| Outpatient visits | Suarez et al., 2016 [[10](#_ENREF_10)] | Peru |  | H65 | Nonsuppurative otitis media | PCV7 (3+1;2+1)/ 3 years | <1 | 26.2 (16.9-34.4) |
|  |  |  |  | H65.0 | Acute serous otitis media | PCV10 (2+1)/ 1 year |  |  |
|  |  |  |  | H65.00 | Acute serous otitis media, unspecified ear |  |  |  |
|  |  |  |  | H65.01 | Acute serous otitis media, right ear |  |  |  |
|  |  |  |  | H65.02 | Acute serous otitis media, left ear |  |  |  |
|  |  |  |  | H65.03 | Acute serous otitis media, bilateral |  |  |  |
|  |  |  |  | H65.04 | Acute serous otitis media, recurrent, right ear |  |  |  |
|  |  |  |  | H65.05 | Acute serous otitis media, recurrent, left ear |  |  |  |
|  |  |  |  | H65.06 | Acute serous otitis media, recurrent, bilateral |  |  |  |
|  |  |  |  | H65.07 | Acute serous otitis media, recurrent, unspecified ear |  |  |  |
|  |  |  |  | H65.1 | Other acute nonsuppurative otitis media |  |  |  |
|  |  |  |  | H65.11 | Acute and subacute allergic otitis media (mucoid) (serous) |  |  |  |
|  |  |  |  | H65.111 | Acute and subacute allergic otitis media (serous), right ear |  |  |  |
|  |  |  |  | H65.112 | Acute and subacute allergic otitis media (serous), left ear |  |  |  |
|  |  |  |  | H65.113 | Acute and subacute allergic otitis media (serous), bilateral |  |  |  |
|  |  |  |  | H65.114 | Acute and subacute allergic otitis media, recur, right ear |  |  |  |
|  |  |  |  | H65.115 | Acute and subacute allergic otitis media, recur, left ear |  |  |  |
|  |  |  |  | H65.116 | Acute and subacute allergic otitis media (serous), recurrent , bilateral |  |  |  |
|  |  |  |  | H65.117 | Acute and subacute allergic otitis media, recurrent, unspecified ear |  |  |  |
|  |  |  |  | H65.119 | Acute and subacute allergic otitis media (serous), unspecified ear |  |  |  |
|  |  |  |  | H65.19 | Other acute nonsuppurative otitis media |  |  |  |
|  |  |  |  | H65.191 | Other acute nonsuppurative otitis media, right ear |  |  |  |
|  |  |  |  | H65.192 | Other acute nonsuppurative otitis media, left ear |  |  |  |
|  |  |  |  | H65.193 | Other acute nonsuppurative otitis media, bilateral |  |  |  |
|  |  |  |  | H65.194 | Other acute nonsuppurative otitis media, recurrent, right ear |  |  |  |
|  |  |  |  | H65.195 | Other acute nonsuppurative otitis media, recurrent, left ear |  |  |  |
|  |  |  |  | H65.196 | Other acute nonsuppurative otitis media, recurrent, bilateral |  |  |  |
|  |  |  |  | H65.197 | Other acute nonsuppurative otitis media recurrent, unspecified ear |  |  |  |
|  |  |  |  | H65.199 | Other acute nonsuppurative otitis media, unspecified ear |  |  |  |
|  |  |  |  | H65.2 | Chronic serous otitis media |  |  |  |
|  |  |  |  | H65.20 | Chronic serous otitis media, unspecified ear |  |  |  |
|  |  |  |  | H65.21 | Chronic serous otitis media, right ear |  |  |  |
|  |  |  |  | H65.22 | Chronic serous otitis media, left ear |  |  |  |
|  |  |  |  | H65.23 | Chronic serous otitis media, bilateral |  |  |  |
|  |  |  |  | H65.3 | Chronic mucoid otitis media |  |  |  |
|  |  |  |  | H65.30 | Chronic mucoid otitis media, unspecified ear |  |  |  |
|  |  |  |  | H65.31 | Chronic mucoid otitis media, right ear |  |  |  |
|  |  |  |  | H65.32 | Chronic mucoid otitis media, left ear |  |  |  |
|  |  |  |  | H65.33 | Chronic mucoid otitis media, bilateral |  |  |  |
|  |  |  |  | H65.4 | Other chronic nonsuppurative otitis media |  |  |  |
|  |  |  |  | H65.41 | Chronic allergic otitis media |  |  |  |
|  |  |  |  | H65.411 | Chronic allergic otitis media, right ear |  |  |  |
|  |  |  |  | H65.412 | Chronic allergic otitis media, left ear |  |  |  |
|  |  |  |  | H65.413 | Chronic allergic otitis media, bilateral |  |  |  |
|  |  |  |  | H65.419 | Chronic allergic otitis media, unspecified ear |  |  |  |
|  |  |  |  | H65.49 | Other chronic nonsuppurative otitis media |  |  |  |
|  |  |  |  | H65.491 | Other chronic nonsuppurative otitis media, right ear |  |  |  |
|  |  |  |  | H65.492 | Other chronic nonsuppurative otitis media, left ear |  |  |  |
|  |  |  |  | H65.493 | Other chronic nonsuppurative otitis media, bilateral |  |  |  |
|  |  |  |  | H65.499 | Other chronic nonsuppurative otitis media, unspecified ear |  |  |  |
|  |  |  |  | H65.9 | Unspecified nonsuppurative otitis media |  |  |  |
|  |  |  |  | H65.90 | Unspecified nonsuppurative otitis media, unspecified ear |  |  |  |
|  |  |  |  | H65.91 | Unspecified nonsuppurative otitis media, right ear |  |  |  |
|  |  |  |  | H65.92 | Unspecified nonsuppurative otitis media, left ear |  |  |  |
|  |  |  |  | H65.93 | Unspecified nonsuppurative otitis media, bilateral |  |  |  |
|  |  |  |  | H66 | Suppurative and unspecified otitis media |  |  |  |
|  |  |  |  | H66.0 | Acute suppurative otitis media |  |  |  |
|  |  |  |  | H66.00 | Acute suppurative otitis media without spontaneous rupture of ear drum |  |  |  |
|  |  |  |  | H66.001 | Acute suppurative otitis media without spontaneous rupture of ear drum, right ear |  |  |  |
|  |  |  |  | H66.002 | Acute suppurative otitis media without spontaneous rupture of ear drum, left ear |  |  |  |
|  |  |  |  | H66.003 | Acute suppurative otitis media without spontaneous rupture of ear drum, bilateral |  |  |  |
|  |  |  |  | H66.004 | Acute suppurative otitis media without spontaneous rupture of ear drum, recurrent, right ear |  |  |  |
|  |  |  |  | H66.005 | Acute suppurative otitis media without spontaneous rupture of ear drum, recurrent, left ear |  |  |  |
|  |  |  |  | H66.006 | Acute suppurative otitis media without spontaneous rupture of ear drum, recurrent, bilateral |  |  |  |
|  |  |  |  | H66.007 | Acute suppurative otitis media without spontaneous rupture of ear drum, recurrent, unspecified ear |  |  |  |
|  |  |  |  | H66.009 | Acute suppurative otitis media without spontaneous rupture of ear drum, unspecified ear |  |  |  |
|  |  |  |  | H66.01 | Acute suppurative otitis media with spontaneous rupture of ear drum |  |  |  |
|  |  |  |  | H66.011 | Acute suppurative otitis media with spontaneous rupture of ear drum, right ear |  |  |  |
|  |  |  |  | H66.012 | Acute suppurative otitis media with spontaneous rupture of ear drum, left ear |  |  |  |
|  |  |  |  | H66.013 | Acute suppurative otitis media with spontaneous rupture of ear drum, bilateral |  |  |  |
|  |  |  |  | H66.014 | Acute suppurative otitis media with spontaneous rupture of ear drum, recurrent, right ear |  |  |  |
|  |  |  |  | H66.015 | Acute suppurative otitis media with spontaneous rupture of ear drum recurrent, left ear |  |  |  |
|  |  |  |  | H66.016 | Acute suppurative otitis media with spontaneous rupture of ear drum recurrent, bilateral |  |  |  |
|  |  |  |  | H66.017 | Acute suppurative otitis media with spontaneous rupture of ear drum recurrent, unspecified ear |  |  |  |
|  |  |  |  | H66.019 | Acute suppurative otitis media with spontaneous rupture of ear drum, unspecified ear |  |  |  |
|  |  |  |  | H66.1 | Chronic tubotympanic suppurative otitis media |  |  |  |
|  |  |  |  | H66.10 | Chronic tubotympanic suppurative otitis media. unspecified |  |  |  |
|  |  |  |  | H66.11 | Chronic tubotympanic suppurative otitis media, right ear |  |  |  |
|  |  |  |  | H66.12 | Chronic tubotympanic suppurative otitis media, left ear |  |  |  |
|  |  |  |  | H66.13 | Chronic tubotympanic suppurative otitis media, bilateral |  |  |  |
|  |  |  |  | H66.2 | Chronic atticoantral suppurative otitis media |  |  |  |
|  |  |  |  | H66.20 | Chronic atticoantral suppurative otitis media, unspecified ear |  |  |  |
|  |  |  |  | H66.21 | Chronic atticoantral suppurative otitis media, right ear |  |  |  |
|  |  |  |  | H66.22 | Chronic atticoantral suppurative otitis media, left ear |  |  |  |
|  |  |  |  | H66.23 | Chronic atticoantral suppurative otitis media, bilateral |  |  |  |
|  |  |  |  | H66.3 | Other chronic suppurative otitis media |  |  |  |
|  |  |  |  | H66.3X | Other chronic suppurative otitis media |  |  |  |
|  |  |  |  | H66.3X1 | Other chronic suppurative otitis media, right ear |  |  |  |
|  |  |  |  | H66.3X2 | Other chronic suppurative otitis media, left ear |  |  |  |
|  |  |  |  | H66.3X3 | Other chronic suppurative otitis media, bilateral |  |  |  |
|  |  |  |  | H66.3X9 | Other chronic suppurative otitis mediam, unspecified ear |  |  |  |
|  |  |  |  | H66.4 | Suppurative otitis media, unspecified |  |  |  |
|  |  |  |  | H66.40 | Other chronic suppurative otitis media, unspecified ear |  |  |  |
|  |  |  |  | H66.41 | Other chronic suppurative otitis media right ear |  |  |  |
|  |  |  |  | H66.42 | Other chronic suppurative otitis media left ear |  |  |  |
|  |  |  |  | H66.43 | Other chronic suppurative otitis media bilateral |  |  |  |
|  |  |  |  | H66.9 | Otitis media, unspecified |  |  |  |
|  |  |  |  | H66.90 | Otitis media, unspecified ear |  |  |  |
|  |  |  |  | H66.91 | Otitis media, right ear |  |  |  |
|  |  |  |  | H66.92 | Otitis media, left ear |  |  |  |
|  |  |  |  | H66.93 | Otitis media, bilateral |  |  |  |
|  |  |  |  | H67 | Otitis media in diseases classified elsewhere |  |  |  |
|  |  |  |  | H67.1 | Otitis media in diseases classified elsewhere, right ear |  |  |  |
|  |  |  |  | H67.2 | Otitis media in diseases classified elsewhere, left ear |  |  |  |
|  |  |  |  | H67.3 | Otitis media in diseases classified elsewhere, bilateral |  |  |  |
|  |  |  |  | H67.9 | Otitis media in diseases classified elsewhere, unspecified ear |  |  |  |
| Outpatient visits | Mackenzie et al., 2009 [[11](#_ENREF_11)] | Tiwi islands, Australia | Moderate or marked bulging of the TM |  |  | PCV7 (3+0) and PPV23  (booster dose)/ 4 years | <2 | 12.0 (13-31) |

^a^ ICD-10 it is not completely equivalent to the source ICD-9.

^b^ Additional reduction after PCV13 introduction.

AOM: Acute Otitis Media; CI: Confidence Interval; ICD: International Classification of Diseases (9th Revision and 10th Revision); NOS: Not Otherwise Specified; OM: Otitis Media; PCV: Pneumococcal Conjugate Vaccine (7, 10 and 13-valent); TM: Tympanic membrane; TMP: Tympanic membrane perforation; US: United States

**References**

1. Grijalva CG, Poehling KA, Nuorti JP, Zhu Y, Martin SW, Edwards KM, et al. National impact of universal childhood immunization with pneumococcal conjugate vaccine on outpatient medical care visits in the United States. Pediatrics. 2006;118(3):865-73. doi: 10.1542/peds.2006-0492. PubMed PMID: 16950975.

2. Poehling KA, Szilagyi PG, Grijalva CG, Martin SW, LaFleur B, Mitchel E, et al. Reduction of frequent otitis media and pressure-equalizing tube insertions in children after introduction of pneumococcal conjugate vaccine. Pediatrics. 2007;119(4):707-15. doi: 10.1542/peds.2006-2138. PubMed PMID: 17403841.

3. Grijalva CG, Nuorti JP, Griffin MR. Antibiotic prescription rates for acute respiratory tract infections in US ambulatory settings. JAMA. 2009;302(7):758-66. doi: 10.1001/jama.2009.1163. PubMed PMID: 19690308; PubMed Central PMCID: PMC4818952.

4. Singleton RJ, Holman RC, Plant R, Yorita KL, Holve S, Paisano EL, et al. Trends in otitis media and myringtomy with tube placement among American Indian/Alaska native children and the US general population of children. Pediatr Infect Dis J. 2009;28(2):102-7. doi: 10.1097/INF.0b013e318188d079. PubMed PMID: 19131901.

5. Wals PD, Carbon M, Sevin E, Deceuninck G, Ouakki M. Reduced physician claims for otitis media after implementation of pneumococcal conjugate vaccine program in the province of Quebec, Canada. Pediatr Infect Dis J. 2009;28(9):e271-5. doi: 10.1097/INF.0b013e3181bad212. PubMed PMID: 19710582.

6. Marom T, Tan A, Wilkinson GS, Pierson KS, Freeman JL, Chonmaitree T. Trends in otitis media-related health care use in the United States, 2001-2011. JAMA Pediatr. 2014;168(1):68-75. doi: 10.1001/jamapediatrics.2013.3924. PubMed PMID: 24276262; PubMed Central PMCID: PMC3947317.

7. Leach AJ, Wigger C, Andrews R, Chatfield M, Smith-Vaughan H, Morris PS. Otitis media in children vaccinated during consecutive 7-valent or 10-valent pneumococcal conjugate vaccination schedules. BMC pediatrics. 2014;14:200. doi: 10.1186/1471-2431-14-200. PubMed PMID: 25109288; PubMed Central PMCID: PMC4149294.

8. Ben-Shimol S, Givon-Lavi N, Leibovitz E, Raiz S, Greenberg D, Dagan R. Near-elimination of otitis media caused by 13-valent pneumococcal conjugate vaccine (PCV) serotypes in southern Israel shortly after sequential introduction of 7-valent/13-valent PCV. Clin Infect Dis. 2014;59(12):1724-32. doi: 10.1093/cid/ciu683. PubMed PMID: 25159581.

9. Lau WC, Murray M, El-Turki A, Saxena S, Ladhani S, Long P, et al. Impact of pneumococcal conjugate vaccines on childhood otitis media in the United Kingdom. Vaccine. 2015;33(39):5072-9. doi: 10.1016/j.vaccine.2015.08.022. PubMed PMID: 26297875.

10. Suarez V, Michel F, Toscano CM, Bierrenbach AL, Gonzales M, Alencar AP, et al. Impact of pneumococcal conjugate vaccine in children morbidity and mortality in Peru: Time series analyses. Vaccine. 2016;34(39):4738-43. doi: 10.1016/j.vaccine.2016.07.027. PubMed PMID: 27521230.

11. Mackenzie GA, Carapetis JR, Leach AJ, Morris PS. Pneumococcal vaccination and otitis media in Australian Aboriginal infants: comparison of two birth cohorts before and after introduction of vaccination. BMC pediatrics. 2009;9:14. doi: 10.1186/1471-2431-9-14. PubMed PMID: 19228431; PubMed Central PMCID: PMC2656498.
